# The Effective Analysis for Blue Honeysuckle Extract in the Treatment of Hepatocellular Carcinoma

**DOI:** 10.1155/2022/9601020

**Published:** 2022-09-29

**Authors:** Chun-Peng Zhang, Wei-Hua Li, Jia-Ren Liu, Guo-Dong Li, Hao-Peng Zhang, Jiu-Feng Wei, Hong-Sheng Chen, Jin-Lu Zhao, Yun-Feng Wang, Qiang Lv, Ming Liu

**Affiliations:** ^1^Department of General Surgery, The Second Affiliated Hospital of Harbin Medical University, Harbin 150081, China; ^2^Department of General Surgery & Bio-Bank of General Surgery, The Fourth Affiliated Hospital of Harbin Medical University, Harbin 150001, China; ^3^Medical Imaging Department, Shenzhen Second People's Hospital-The First Affiliated Hospital of Shenzhen University Health Science Center, Shenzhen 518035, China; ^4^Department of Laboratory Diagnosis, The Fourth Affiliated Hospital of Harbin Medical University, Harbin 150001, China; ^5^Department of Hepatobiliary and Pancreatic Surgery, Heilongjiang Provincial Tumor Hospital, Harbin 150081, China

## Abstract

To further determine how BHE affected the growth of HCC cells, the proportion of each cell cycle phase was explored in HCC cells by flow cytometry. Blue honeysuckle (*Lonicera caerulea* L.) is a species of bush that grows in eastern Russia. Blue honeysuckle extract (BHE) is rich in bioactive phytochemicals which can inhibit the proliferation of tumor cells. The mechanism underlying the anticancer activity of BHE in primary liver cancer is poorly understood. The purpose of this study was to evaluate the growth inhibition mechanism of bioactive substances from blue honeysuckle on hepatocellular carcinoma (HCC) cells and to explore its protein and gene targets. The compounds in BHE were determined by high-performance liquid chromatography (HPLC) and liquid chromatography-mass spectrometry (LC-MS). Cell counting kit-8 (CCK8) assay was used to evaluate the effects of BHE on HCC cell proliferation, and flow cytometry assay (FCA) was used to determine how BHE arrested the proportion of each cell cycle phase in HCC cells. Western blot (WB) was performed to determine the expression of cell cycle-related proteins in HCC cells treated with different concentrations of BHE. The xenograft tumor animal models were established by HCC cell implantation. The results showed that cyanidin-3-o-glucoside and cyanidin-3-o-sophoroside which are the main biologically active components were detected in BHE. BHE is highly effective in inhibiting the proliferation of HCC cells by arresting the HCC cell cycle in the G2/M phase. BHE also downregulated the expression of conventional or classical dendritic cells-2 (cDC2) and cyclin B1 by promoting the expression of myelin transcription factor 1 (MyT1) in HCC cells. The weight and volume of xenografts were significantly decreased in the BHE treated groups when compared to the control group. BHE increased the expression of MyT1 in xenograft tissues. These findings showed that blue honeysuckle extract inhibits proliferation *in vivo* and *in vitro* by downregulating the expression of cDC2 and cyclin B1 and upregulating the expression of MyT1 in HCC cells.

## 1. Introduction

Hepatocellular carcinoma (HCC) is the most common primary malignant tumor of the liver. It is the sixth most common type of cancer and constitutes the fourth major cancer-related mortality worldwide in 2018. There are about 841,000 new HCC cases and 782,000 HCC-related deaths annually [1]. Worse yet is that both incidence and mortality of HCC show an upward trend worldwide in recent years, with a higher prevalence in Asia, accounting for nearly 70% of new cases worldwide [2]. 55% of all HCC cases are diagnosed in China [3, 4]. The early stage of HCC tends to be asymptomatic, so the disease may have progressed to an advanced stage by the time of diagnosis. The therapeutic effects of current treatment approaches for HCC are also compromised by high relapse rates, leading to the poor long-term survival of HCC patients [5]. Surgery is a treatment mainstay, but its invasiveness may affect the postoperative quality of life and is associated with local recurrences. Thus, early chemoprevention, detection, diagnosis, and treatment are of great significance to enhance the prognosis of HCC patients [6, 7].

Plant antioxidants play an important role in mitigating the impact of oxidative stress on life genetic materials and cell structures, reducing the damage of chronic diseases, and facilitating the maintenance of the normal metabolism of tissue cells and various biological characteristics. These biological activities include oxidative damage attenuation, alleviation of radiation damage and inflammation, cardiomyocyte protection, bacteriostasis, gastroprotection [8, 9], hepatoprotection [10, 11], and other relevant properties [12, 13]. The chemoprophylaxis of blue honeysuckle stems from its high concentration of highly active phytochemicals and anthocyanins that have shown significant benefits against various human diseases [14]. In the past few decades, anthocyanins have captured great attention in the treatment of diseases including infections, neurological diseases, cancer [14–18], and diabetes [19, 20]. Blue honeysuckle (*Lonicera caerulea* L.), a fruit grown worldwide, is a nutrient-rich forest berry. The fruit of the blue honeysuckle is rich in a variety of vitamins, minerals, amino acids, flavonoids, carbohydrates, polyols, organic acids, and other bioactive substances [21, 22]. The health benefits of blue honeysuckle have been widely explored due to its rich bioactive components. These components mainly include phenolic acid, anthocyanin, and flavonoids, and vitamin B, magnesium, phosphorus, calcium, and potassium are secondary blue honeysuckle components [23, 24]. Blue honeysuckle contains great amounts of polyphenolic compounds [25, 26], such as anthocyanins, pelargonidin, peonidin, catechol, flavanols, and chlorogenic acid [27]. The total anthocyanin contents in blue honeysuckle are higher than that in other fruits [28]. Anthocyanins are the main polyphenolic compounds in blue honeysuckle [29], and the anthocyanin-3-glycoside is the main form of anthocyanin present in blue honeysuckle [24, 30, 31]. The blue honeysuckle contains higher contents of cyanidin-3-glucoside than other berries, including blueberries, blackberries, and raspberries [30]. Anthocyanins are mainly found in the peels of fruits, with a broad range of well-recognized biological activities including antimicrobial, anticancer, and anti-inflammatory activities [24]. Few studies have investigated the antitumor mechanism of fresh blue honeysuckle at the protein and molecular levels. In particular, efficient methods to extract bioactive substances from fresh blue honeysuckle and clarification of the molecular mechanisms underlying its antitumor activity have been sparingly explored. Thus, the aim of this study was to investigate the effects of blue honeysuckle extracts (BHE) on the proliferation of HCC cells by exploring its anti-neoplastic action and its molecular protein mechanism, which may provide a basis for the use of BHE as chemoprophylaxis and chemotherapeutic agents for primary liver cancer.

## 2. Materials and Methods

### 2.1. The Extraction of Fresh Blue Honeysuckle

Fully-ripe fresh blue honeysuckle was purchased from Greenfield Berries Ltd., Shangzhi City, Heilongjiang Province. Blue honeysuckles (200 g) were ground with 600 ml of 80% alcohol in a rough homogenate machine for 10 min, and the procedure was performed thrice. The crude blended samples were further mixed using a high rotary cutting homogenizer in an ice bath for 5 min. The homogenates were filtered through a Buchner funnel and vacuum filtered through three layers of Whatman#2 filter papers. The filtrate was rotary evaporated at 40°C for approximately 90 min in an evaporator to remove 95% of the solvent. Three replicates of blue honeysuckle extracts (BHE) were stored in aliquots at −80°C until required for further use.

### 2.2. The Content of Main Compounds in BHE by HPLC

Quantitative analysis of cyanidin-3-o-glucoside and cyanidin-3-o-sophoroside in BHE was performed on High-Performance Liquid Chromatography (HPLC) Surveyor (Thermo Fisher Scientific, Waltham, MA, USA) with a photo-diode array (PDA) detector scanning from 250 to 350 nm. The optimized mobile phases used were 0.1% phosphoric acid in ultrapure water (*A*) and acetonitrile (*B*), and the elution time was 30 min for equal concentration degree (*A*)/(*B*) (90 : 10). Chromatography was carried out at 30°C using Supersil ODS2 chromatographic column (4.6 mm × 250 mm, 5 *μ*m). The chromatograms were recorded at 280 nm, and the 20 *μ*L of injection was applied to the column.

### 2.3. Identification of Fresh BHE by LC-MS

The sample was analyzed by the AB Sciex 5600-Triple-Tof system, which consisted of surveyor autosampler, quaternary pump, online degassing machine, and electrospray ionization (ESI) with positive ionization mode. The flow-through analytical cell used Waters UPLC HSS T3 column (2.1 mm × 150 mm, 1.8 *μ*m) and HSS T3 column (2.1 mm × 5 mm, 1.8 *μ*m) with column temperature of 35°C, sample room temperature of 10°C, and the injection volume of 5 *μ*L. The optimized mobile phases used were 0.1% formic acid in water (*A*) and 0.1% formic acid in acetonitrile (*B*) with gradient elution from 0 to 20 min at a flow rate of 0.3 ml/min. The ionization temperature was increased to 350°C, with a nebulizer and auxiliary gas at a pressure of 50 psi, and the capillary voltage was controlled at 4500 V.

### 2.4. Animal Treatment

Four-week-old athymic BALB/c female nude mice (weight 12∼16 g) were purchased from Beijing Weitonglihua Laboratory Animal Technology Co. Ltd (Beijing, China) and acclimated for 7 days in the animal room. During the study, the mice were fed with sterilized food and water and were housed in a barrier environment under the standard light/dark cycle (12 h light, 12 h dark). The mice were maintained under standard conditions and cared for as per the institutional guidelines and ethical regulations of the National Cancer Institute of China. All protocols of animals were approved by the Committee of Ethics from Harbin Medical University (Harbin, China).

HepG2 cells or Huh7 cells (5 × 10^6^) mixed with Matrigel (3 : 1) were placed subcutaneously on the right ventral dorsum of five-week-old athymic BALB/c female nude mice for a 7-days-of-growth to reach a tumor diameter of 5 mm. The mice with xenografts were randomly divided into 4 groups (5 mice/group): (I) mice received 0.9% normal saline daily by gavage as the control group; (II) mice received 7 g/kg/d of BHE (equivalent to 7 g fresh blue honeysuckle) by gavage; (III) mice received 40 mg/kg/d of anthocyanins by gavage; and (IV) mice received 30 mg/kg of intraperitoneal injection of 5-Fu alternately by gavage. The body weight and volume of the tumor were measured every 4 days. The activity of mice was also closely monitored. After 36 days of treatment, tumor xenografts were excised, collected, and weighed. Parts of xenografts were fixed for immunohistochemistry, and the others were frozen at 80°C for Western blot.

### 2.5. Cell Culture

HepG2 and Huh7 cells were donated by the Key Laboratory of Surgery Ministry, Harbin Medical University. Cells were grown in Dulbecco's modified Eagle's medium (DMEM) containing 10% FBS (fetal bovine serum, v/v), 100 units/ml penicillin, and 100 *μ*g/ml at 37°C in a humidified incubator with 5% CO_2_. Cells were sub-cultured every 2 days at 80–90% confluence for experiments.

### 2.6. Cell Viability

The cell viability of BHE was assessed in HepG2 and Huh7 using the CCK8 (Dojindo Laboratories, Kumamoto, Japan) as in a previous study [32]. Cells were inoculated at a density of 3 × 103 cells/well in a 96-well plate and cultured in medium added with different BHE concentrations (0, 5, 10,15, 20, 30, or 40 mg/ml), anthocyanins (150 *μ*g/ml) (Harbin, China), and 5-FU (10 *μ*g/ml) (Sigma, USA) at 37°C for 24, 48, 72, or 96 h. Anthocyanins (150 *μ*g/ml) and 5-FU (10 *μ*g/ml) were used as the positive controls to evaluate the antitumor efficacy of blue honeysuckle extract from multiple perspectives. After treatment, 10 *μ*L of CCK8 was added to each well and the cells were cultured for 2 h at 37°C. The optical density (OD) of each well was measured at 450 nm using a microplate reader (Bio-Rad Laboratories, Hercules, CA, USA). Cell viability was calculated using the following formula. Relative percentage of cell viability = (OD of the experimental group/OD of the control group) × 100%. The assay was performed in triplicate.

### 2.7. Cell Cycle

The concentrations of BHE were selected for flow cytometry based on the results from CCK8 assay. The cells were inoculated in a culture flask, treated with different concentrations of BHE (0, 5, 10, or 20 mg/ml), anthocyanins (150 *μ*g/ml), and 5 FU (10 *μ*g/ml) at 5% CO_2_ saturated humidity incubator at 37°C. After 48 h, cells were seeded at 1 × 10^6^ cells/well and harvested by trypsinization for cell cycle analysis. After being rinsed with phosphate buffered saline (PBS), the cells were fixed with 1 ml of ice-cold 70% ethanol at −20°C overnight and were centrifuged (2,000*g*) at 4°C for 10 min. Cell pellets were collected, rinsed with PBS, and incubated in a 0.4 ml staining solution (PI: RNaseA, 5 : 1) for 30 min at 37°C, followed by fluorescence-activated cell sorting (FACS) using a FACSCalibur (FACSort, Becton-Dickinson, CA, USA) as in the previous study [33].

### 2.8. Western Blot

Western blot was performed to determine the levels of cyclin B1, cDC2, and MyT1 proteins as in the previous studies [34–36]. Cells were treated with different concentrations of BHE (0, 5, 10, or 20 mg/ml), anthocyanins (150 *μ*g/ml), and 5 FU (10 *μ*g/ml) for 48 h, rinsed twice with ice-cold PBS, and harvested by scraping. Total cellular protein was extracted using RIPA buffer (Cell Signaling Technology, MA, USA) with phenylmethane sulfonyl fluoride in the 1× proteinase inhibitor cocktail (Roche, Basel, Switzerland) per the manufacturer's instructions. Frozen xenograft tissues were soaked and lysed in cold RIPA buffer (Cell Signaling Technology) with phenyl methane sulfonyl fluoride in the 1× proteinase inhibitor cocktail (Roche, Basel) on ice. After stirring, the cells were homogenized with a high-rotation emulsifying homogenizer for 5 min to break the walls. The cells were then centrifuged (14000*g*, 4°C) for 10 min to obtain the supernatant. Total protein concentrations were determined using the BCA Protein Assay Kit (Beyotime, Shanghai, China). Proteins (30 *μ*g) were separated using SDS-polyacrylamide gel electrophoresis (SDS-PAGE) and transferred onto polyvinylidene difluoride (PVDF) membranes. The membranes were blocked with 5% nonfat milk in PBS plus 0.05% Tween 20 (PBST) for 1 h at room temperature. Membranes were then incubated with primary antibodies against cyclin B1 (1 : 1000, San Ying Biotechnology, Wuhan, China), cDC2 (1 : 1000, San Ying Biotechnology, Wuhan, China), MyT1 (1 : 1000, San Ying Biotechnology, Wuhan, China), and *β*-actin (1 : 1000, San Ying Biotechnology, Wuhan, China) at 4°C overnight. Membranes were rinsed with PBST 3 times and then incubated with horseradish peroxidase-conjugated secondary antibodies for 2 h at room temperature. After being rinsed with PBST three times again, the enhanced chemiluminescence (ECL) reagents (Beyotime, Shanghai, China) were uniformly applied to the PVDF membrane according to the manufacturer's protocol for color development.

### 2.9. Immunohistochemistry

The expression of cyclin B1, cDC2, and MyT1 in xenografts was determined by immunohistochemistry as in the previous studies [37, 38]. Briefly, the sections of paraffin-embedded xenograft tumor tissue were dewaxed by xylene and hydrated with a graded ethanol solution. The sections were rinsed using PBS (pH 7.4) three times. The antigen retrieval was performed using the sodium citrate buffer (pH 6.0) at 95–100°C for 30 min, and the sections were cooled at room temperature. Then, the sections were pretreated in a 3% (v/v) hydrogen peroxide solution for 10 min to block endogenous peroxidase activity. After being rinsed with PBS three times, nonspecific binding serum was added to the sections for 10 min. Anti-cyclin B1, anti-cDC2, and anti-MyT1 antibodies at a dilution of 1 : 100 were added to the sections and incubated at 4°C overnight. After being rinsed with PBS three times, the sections were incubated with biotinylated secondary antibodies (Zhongshanjinqiao Biotechnology Inc, China) for 30 min at 37°C. 3,3-diaminobenzidine (DAB) solution was used for staining, and hematoxylin was used for counterstaining. The expression of cyclin B1, cDC2, and MyT1 was determined by light microscopy with a digital camera.

### 2.10. Statistical Analyses

Data are expressed by means ± standard deviation (S.D.). Difference analysis was performed using GraphPad Prism 8.0 (GraphPad Software, USA). Student's *t*-test and variance analyses were performed to analyze the significance of differences with inter-group comparisons. Statistically significant results were defined as *P* < 0.05.

## 3. Results

### 3.1. The Components Identified in BHE

**Figure 1:**
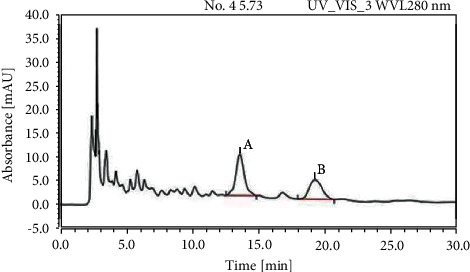
Contents of cyanidin-3-o-sophoroside and cyanidin-3-o-glucoside in blue honeysuckle extract. The contents of *A* and *B* were determined in blue honeysuckle extract (BHE) by HPLC. *A* for cyanidin-3-o-sophoroside and *B* for cyanidin-3-o-glucoside.

**Figure 2:**
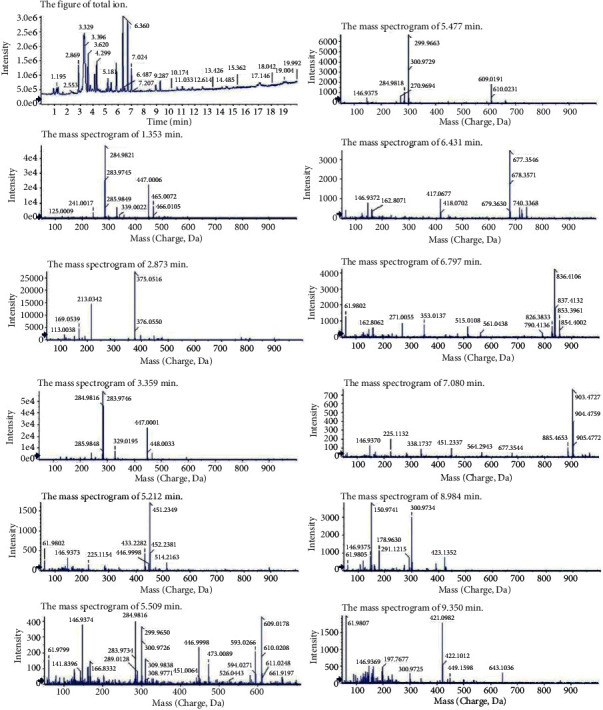
The total ion chromatogram and mass spectrograms of BHE. The compounds of BHE were identified by HPLC and LC-MS.

As shown in Figure 1, cyanidin-3-o-glucoside and cyanidin-3-o-sophoroside were identified in BHE by HPLC. The average contents of cyanidin-3-o-glucoside and cyanidin-3-o-sophoroside are shown in Table 1. There were 0.544 ± 0.012 mg and 0.578 ± 0.011 mg per 100 g of fresh blue honeysuckle for cyanidin-3-o-glucoside and cyanidin-3-o-sophoroside, respectively. Other components were also identified by LC-MS (Table 2), and several unknown components were found in BHE (Figure 2).

### 3.2. BHE Reduced the Proliferation of HCC Cells

**Figure 3:**
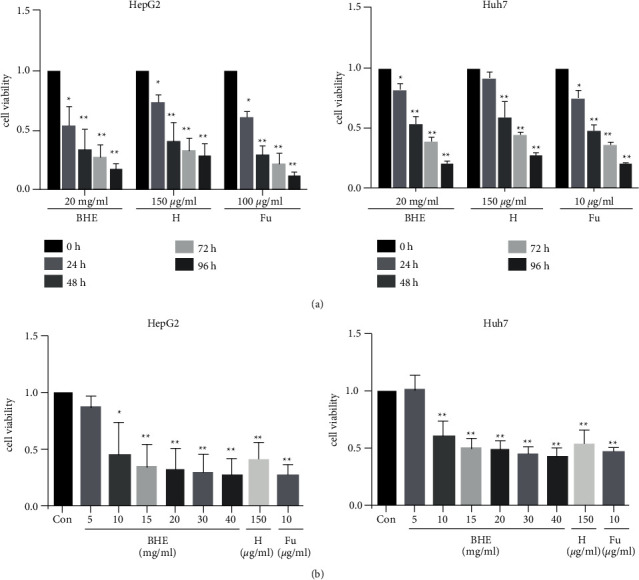
The cell viability of BHE in HepG2 and Huh7 cells. HepG2 cells or Huh7 cells were treated with 20 mg/ml of BHE, 150 *μ*g/ml of anthocyanins, or 10 *μ*g/ml of 5 FU for 24, 48, 72, or 96 h (*n* = 6). The cell viability was determined by the CCK8 assay. (a) The cell viability of BHE in HepG2 and Huh7 cells for 24, 48, 72, or 96 h. (b) The cell viability of BHE in HepG2 and Huh7 cells for 48 h. The data are expressed as mean ± standard deviation (S.D.). ^*∗*^*P* < 0.05, ^*∗∗*^*P* < 0.01, when compared to the negative control group.

To assess the effect of BHE on antitumor activity, a CCK8 assay was performed to determine the cell viability of HepG2 cells and Huh7 cells. BHE significantly inhibited the proliferation of HepG2 cells and Huh7 cells in a dose- and time-dependent manner (*P* < 0.05 and *P* < 0.01) (Figures 3(a) and 3(b)). The 50% inhibitory concentrations (IC_50_) were 52.79 and 41.04 mg/ml in HepG2 and Huh7 cells, respectively. The positive controls, i.e., anthocyanins (150 *μ*g/ml) and 5 FU (10 *μ*g/ml), also showed potent inhibition of proliferation in both HepG2 and Huh7 cells (Figure 3).

### 3.3. BHE Arrested the Proportion of Each Cell Cycle Phase in HCC Cells

**Figure 4:**
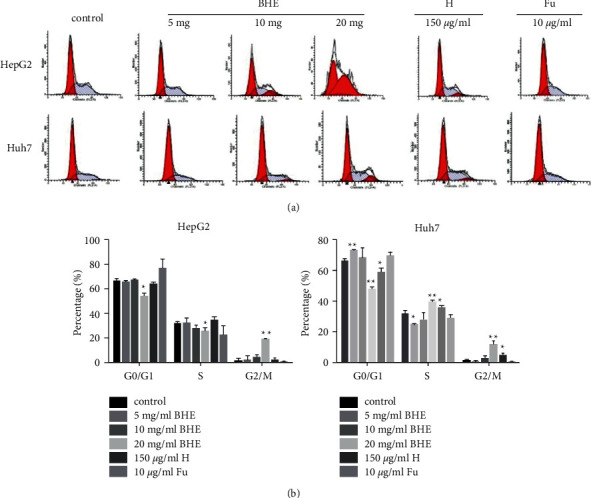
(a) The distribution of cell cycle in HepG2 and Huh7 cells. HepG2 and Huh7 cells were treated with different concentrations of BHE, 150 *μ*g/ml of anthocyanins, or 10 *μ*g/ml of 5 FU for 48 h. (b) The distribution of the cell cycle was determined in HepG2 and Huh7 cells by flow cytometry (*n* = 3). ^*∗*^*P* < 0.05, ^*∗∗*^*P* < 0.01, when compared to the negative control group.

To further determine how BHE affected the growth of HCC cells, the proportion of each cell cycle phase was explored in HCC cells by flow cytometry as shown in Figure 4. BHE could affect the distribution of the cell cycle in HepG2 and Huh7 cells. After being cultivated with BHE at dose of 0, 5, 10, or 20 mg/ml or 150 *μ*g/ml of anthocyanin for 48 h, the proportion of cell cycle at the G2/M phase increased gradually from 1.33 ± 2.16% to 2.28 ± 0.04%, 4.39 ± 0.02%, 12.66 ± 0.11%, and 2.11 ± 0.02% in HepG2 cells. However, the share of cell cycle at the G2/M phase changed from 1.57 ± 0.01% to 0.71 ± 0.01%, 2.93 ± 0.019%, 11.68 ± 0.03%, and 4.89 ± 0.02% in Huh7 cells (Figure 4).

### 3.4. BHE Affected the Expression of Cell Cycle-Related Protein in HCC Cells

**Figure 5:**
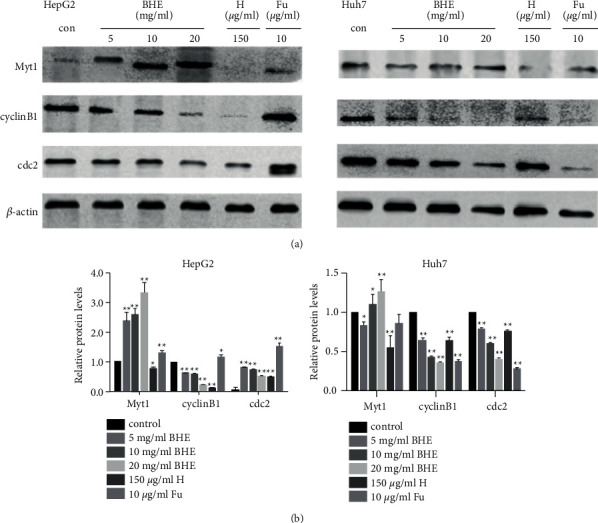
(a) The expression of cyclin B1, cDC2, and MyT1 proteins in HepG2 and Huh7 cells. HepG2 and Huh7 cells were treated with different concentrations of BHE, 150 *μ*g/ml of anthocyanins, or 10 *μ*g/ml of 5 FU for 48 h. (b) The expression of cyclin B1, cDC2, and MyT1 proteins was explored in HepG2 and Huh7 cells by Western blot. ^*∗*^*P* < 0.05, ^*∗∗*^*P* < 0.01, when compared to the negative control group.

To explore the molecular mechanism of the BHE-arrested cell cycle at the G2/M phase, the expression of protein related to the cell cycle was examined in HepG2 and Huh7 cells by Western blot. After being cultivated with BHE (0, 5, 10, and 20 mg/ml), anthocyanin (150 *μ*g/ml), or 5 FU (10 *μ*g/ml) for 48 h, the expression of cyclin B1, cDC2, and MyT1 proteins was examined. The results are shown in Figure 5. BHE at the concentration of 20 mg/ml significantly upregulated the expression of MyT1 and downregulated the expression of cyclin B1 and cDC2 in both HepG2 and Huh7 cells (*P* < 0.05 and *P* < 0.01). However, the expression of cyclin B1 and cDC2 differed in HepG2 and Huh7 cells after treatment with anthocyanins or 5 FU. Anthocyanins significantly downregulated the expression of MyT1, cyclin B1, and cDC2 in both cell lines. 5 FU significantly downregulated the expression of MyT1, cyclin B1, and cDC2 in Huh7 cells and showed an opposite expression in HepG2 cells.

### 3.5. BHE Inhibited Growth of Xenografts in a Model of Mice

**Figure 6:**
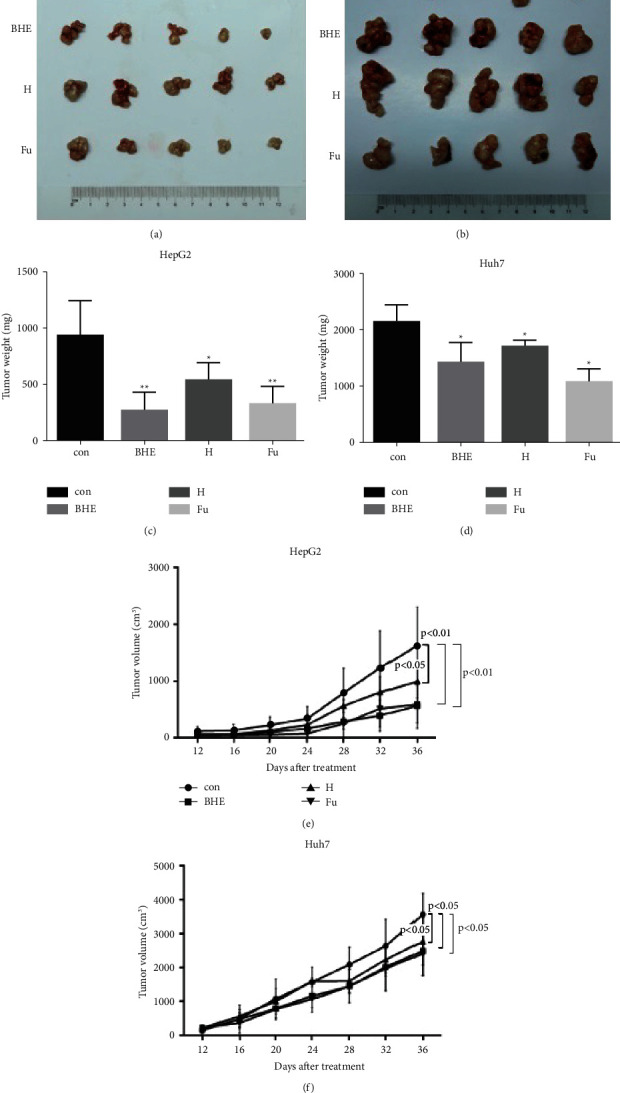
BHE inhibited the growth of xenografts in the model of nude mice. The BALB/c female nude mice were implanted with HepG2 cells or Huh cells for 7 days. The mice with xenografts were randomly divided into the positive control group, 7 g/kg/d of BHE (equivalent to 7 g fresh blue honeysuckle) group, 40 mg/kg/d of anthocyanins group, and 30 mg/kg of 5 Fu group (5 mice/group). The tumor sizes were measured every 4 days. After treatment for 36 days, xenografts were collected and weighted. (a) The size of HepG2 cell xenografts in nude mice. (b) The size of Huh cell xenografts in nude mice. (c) The weight of tumor in HepG2 cell xenografts of nude mice. (d) The weight of tumor in Huh cell xenografts of nude mice. (e) The changes of tumor volume in nude mice (HepG2 cells). (f) The changes of tumor volume in nude mice (Huh cells). ^*∗*^*P* < 0.05, ^*∗∗*^*P* < 0.01, when compared to the positive control group.

To further investigate the effects of BHE on anticancer activity, athymic BALB/c female nude mice were implanted with HepG2 and Huh7 cells. As shown in Figure 6, BHE significantly decreased the growth of xenografts in both HepG2 and Huh7 cells. BHE decreased both the weight of the tumor in the experimental termination and the volume of the tumor in a time-dependent manner (*P* < 0.05 and *P* < 0.01) (Figure 6). The weight of xenografts in the BHE group was 278 ± 105 g and 1432 ± 212 g in HepG2 and Huh7 cells, respectively, when compared to the control group (936 ± 178 g and 2140 ± 187 g) (*P* < 0.05 and *P* < 0.01). The final volumes of xenografts in the BHE group were 568 ± 242 mm^3^ and 2499 ± 545 mm^3^ in HepG2 and Huh7 cells, respectively, when compared to the control group (1629 ± 529 mm^3^ and 3575 ± 485 mm^3^) (*P* < 0.05 and *P* < 0.01). The weight of the mice in the BHE group did not differ from the control group (data not shown). In addition, anthocyanins and 5 FU also showed an inhibitory effect on the growth of xenografts in this mouse model.

### 3.6. BHE Affected the Expression of MyT1, Cyclin B1, and cDC2 in Xenograft Tissues

**Figure 7:**
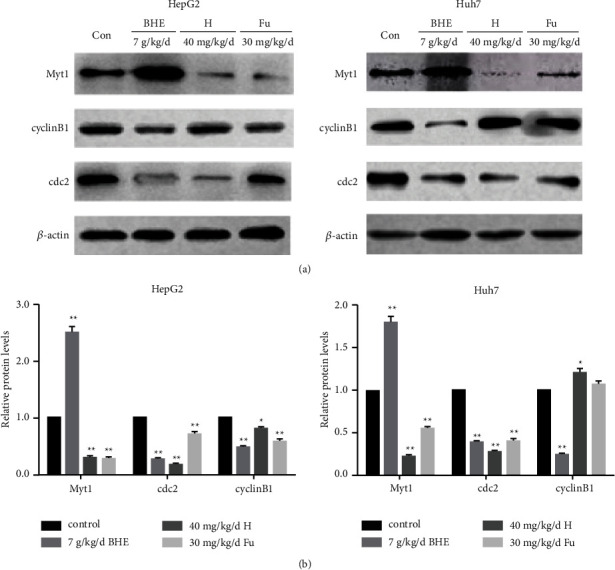
(a) The expression of cyclin B1, cDC2, and MyT1 proteins in xenograft tissues. (b) The expression of cyclin B1, cDC2, and MyT1 proteins in xenograft tissues analyzed by Western blot. ^*∗*^*P* < 0.05, ^*∗∗*^*P* < 0.01, when compared to the positive control group.

**Figure 8:**
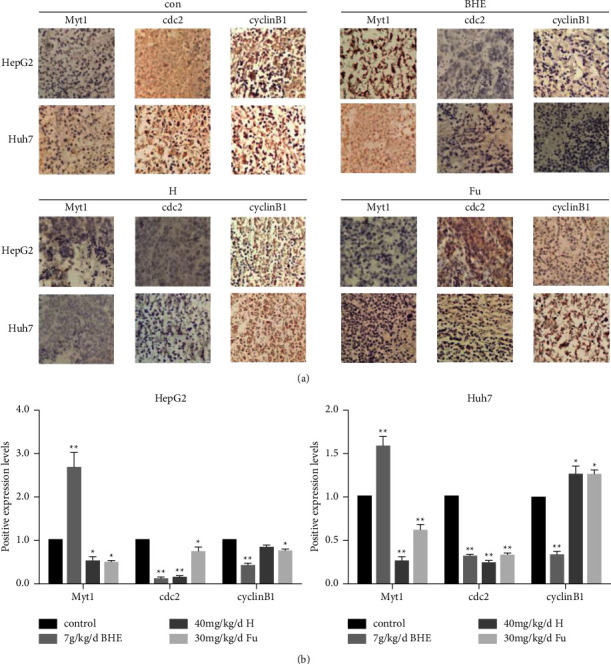
(a) The expression of cyclin B1, cDC2, and MyT1 proteins in xenograft tissues. (b) The expression of cyclin B1, cDC2, and MyT1 proteins in xenograft tissues analyzed by immunohistochemistry. ^*∗*^*P* < 0.05, ^*∗∗*^*P* < 0.01, when compared to the positive control group.

To further investigate the possible mechanism of BHE inhibiting hepatocellular carcinoma growth *in vivo*, the expression of cyclin B1, cDC2, and MyT1 proteins was determined in xenograft tissues using Western blot and immunohistochemistry. The results are shown in Figures 7 and 8. BHE significantly decreased the expression of cyclin B1 and cDC2 and significantly increased the expression of MyT1 in xenograft tissues when compared to the group (*P* < 0.05 and *P* < 0.01).

## 4. Discussion

Cancer is one of the major causes of disease-related mortality worldwide. The pathogenesis of cancer is complex and involves alterations in cancer-related genes and tumor suppressor genes. Some cytokines also play roles in the occurrence and development of cancer. The actions of genes and cytokines lead to the malignant proliferation of cells, so inhibition of the proliferation of malignant cells is the cornerstone of tumor therapy. Our findings indicated that BHE could inhibit the proliferation of HCC cells in a dose-dependent manner.

Currently, the most common methods used to treat malignant tumors are surgery, chemotherapy, and radiotherapy. However, these approaches are associated with multiple toxic side effects, high recurrent rates, and drug resistance. Bioactive components from natural plants for antitumor purposes that cause fewer side effects might be another potentially effective approach for tumor therapy. The blue honeysuckle is rich in nutrients and bioactive substances, especially natural pigment anthocyanins, oxidized scavenger flavonoids, and plant fungicide organic phenolic acids. These bioactive components have strong antioxidant activities [22, 27]. Phytochemicals that are rich in BHE play an important role in inhibiting the development of different cancers, including colon [39–41], oral [42], lung [43], prostate [44], breast [45, 46], and skin cancer [47]. In the present study, the main biologically active components determined by HPLC in BHE were cyanidin-3-o-glucoside and cyanidin-3-o-sophoroside. The contents of cyanidin-3-o-glucoside and cyanidin-3-o-sophoroside of BHE were 0.544 ± 0.012 mg and 0.578 ± 0.011 mg per 100 g of fresh blue honeysuckle. Moreover, the compounds in BHE identified by LC-MS were cyanidin-3-o-glucoside, cyanidin-3-o-sophoroside, ellagic acid, and some unknown components.

In this study, BHE inhibited cell proliferation of HCC by arresting the cell cycle at the G2/M phase in HepG2 and Huh7 cells. The expression of the cDC2/cyclin B1 pathway in HepG2 and Huh7 cells was also determined. The cDC2 is the most important cell cycle gene in hematological malignant tumors [48, 49]. cDC2 inhibits the progression of the cell cycle and potentially induces apoptosis. The activation or over-expression of cDC2 promotes cell proliferation and imbalances the cell proliferation and apoptosis, which suggests the role of cDC2 in multiple tumorigenesis processes. Additionally, cyclin B-associated cDC2 regulates the G2/M phase [50]. The results of the present study suggested that BHE arrested the progression of the cell cycle at the G2/M phase by altering levels of MyT1, cyclin B1, and cDC2 in HepG2 and Huh7 cells.

A model of nude mice was established in this study, and the results showed that BHE inhibited the growth of xenografts and affected the expression of MyT1, cyclin B1, and cDC2. These findings confirmed that BHE inhibited the proliferation of HCC by arresting the cell cycle of HCC and affecting the expression of proteins related to the cell cycle. However, further clinical trials are required for the clinical application of blue honeysuckle extract.

## 5. Conclusion

BHE inhibits the cyclin B1/cDC2 signaling pathways to achieve antitumor effects, which demonstrates its great potential as a new anticancer drug for the treatment of patients with HCC and provides a theoretical basis for the further development and utilization of blue honeysuckle in tumor therapy.

## Figures and Tables

**Table 1:** The contents of cyanidin-3-o-sophoroside and cyanidin-3-o-glucoside in samples of blue honeysuckle (mg/g fresh weight).

Content	Cyanidin-3-O-sophoroside	Cyanidin-3-O-glucoside
1	0.568	0.543
2	0.577	0.556
3	0.589	0.533
Average	0.578 ± 0.011	0.544 ± 0.012

**Table 2:** The main components identified in BHE by LC-MS.

No.	tR (min)	[M-H]	Fragment ions	Tentatively identification
1	1.353	447.0	339.0 283.9 (M-Glc-Glc)	Cyanidin-3-O-glucoside isomer
2	2.873	375.1	213.0 (M-Glc), 168.0	—
3	3.359	447.0	284.9 (M-Rha-glc)	Cyanidin-3-O-glucoside
4	5.212	451.2	341.0, 162.8,61.9	—
5	5.509	609.0	564.3, 300.9, 61.9	Cyanidin-3-O-sophoroside
6	5.477	609.0	412.9, 315.0, 277.2	Cyanidin-3-O-sophoroside isomer
7	6.431	677.3	563.3, 451.2, 337.9, 225.1	—
8	6.797	791.1	563.3, 451.2, 337.9, 225.1, 146.9	—
9	7.080	903.3	695.3, 563.3, 451.2, 337.9, 225.1, 146.9	—
10	8.984	301	151.9, 61.9	Ellagic acid
11	9.350	421.0	197.7, 61.9	—

## Data Availability

All data generated or analyzed during this study are included in this published article.

## References

[1] Bray F., Ferlay J., Soerjomataram I., Siegel R. L., Torre L. A., Jemal A. (2018). Global cancer statistics 2018: GLOBOCAN estimates of incidence and mortality worldwide for 36 cancers in 185 countries. *CA: A Cancer Journal for Clinicians*.

[2] Ferlay J., Colombet M., Soerjomataram I. (2019). Estimating the global cancer incidence and mortality in 2018: GLOBOCAN sources and methods. *International Journal of Cancer*.

[3] Rowe J., Ghouri Y., Mian I. (2017). Review of hepatocellular carcinoma: epidemiology, etiology, and carcinogenesis. *Journal of Carcinogenesis*.

[4] Zhu R. X., Seto W. K., Lai C. L., Yuen M. F. (2016). Epidemiology of hepatocellular carcinoma in the asia-pacific region. *Gut Liver*.

[5] Villanueva A., Llovet J. M. (2011). Targeted therapies for hepatocellular carcinoma. *Gastroenterology*.

[6] Colditz G. A., Wei E. K. (2012). Preventability of cancer: the relative contributions of biologic and social and physical environmental determinants of cancer mortality. *Annual Review of Public Health*.

[7] Steward W. P., Brown K. (2013). Cancer chemoprevention: a rapidly evolving field. *British Journal of Cancer*.

[8] Liu J. R., Dong H. W., Sun X. R. (2009). Effects of beta-ionone on mammary carcinogenesis and antioxidant status in rats treated with DMBA. *Nutrition and Cancer*.

[9] Liu J. R., Sun X. R., Dong H. W. (2008). *β*-ionone suppresses mammary carcinogenesis, proliferative activity and induces apoptosis in the mammary gland of the sprague-dawley rat. *International Journal of Cancer*.

[10] Liu Y., Liu M., Li B. (2010). Fresh raspberry phytochemical extract inhibits hepatic lesion in a Wistar rat model. *Nutrition and Metabolism*.

[11] Chen H. S., Liu M., Shi L. J. (2011). Effects of raspberry phytochemical extract on cell proliferation, apoptosis, and serum proteomics in a rat model. *Journal of Food Science*.

[12] Vostalova J., Galandakova A., Palikova I. (2013). *Lonicera caerulea* fruits reduce UVA-induced damage in hairless mice. *Journal of Photochemistry and Photobiology B: Biology*.

[13] Xie C., Kang J., Ferguson M. E., Nagarajan S., Badger T. M., Wu X. (2011). Blueberries reduce pro-inflammatory cytokine TNF-*α* and IL-6 production in mouse macrophages by inhibiting NF-*κ*B activation and the MAPK pathway. *Molecular Nutrition & Food Research*.

[14] Zafra-Stone S., Yasmin T., Bagchi M., Chatterjee A., Vinson J. A., Bagchi D. (2007). Berry anthocyanins as novel antioxidants in human health and disease prevention. *Molecular Nutrition & Food Research*.

[15] Kim A. J., Park S. (2006). Mulberry extract supplements ameliorate the inflammation-related hematological parameters in carrageenan-induced arthritic rats. *Journal of Medicinal Food*.

[16] Shin W. H., Park S. J., Kim E. J. (2006). Protective effect of anthocyanins in middle cerebral artery occlusion and reperfusion model of cerebral ischemia in rats. *Life Sciences*.

[17] Tarozzi A., Morroni F., Hrelia S. (2007). Neuroprotective effects of anthocyanins and their in vivo metabolites in SH-SY5Y cells. *Neuroscience Letters*.

[18] Cohen-Boulakia F., Valensi P. E., Boulahdour H. (2000). In vivo sequential study of skeletal muscle capillary permeability in diabetic rats: effect of anthocyanosides. *Metabolism*.

[19] Jankowski A., Jankowska B., Niedworok J. (2000). The effect of anthocyanin dye from grapes on experimental diabetes. *Folia Medica Cracoviensia*.

[20] Pergola C., Rossi A., Dugo P., Cuzzocrea S., Sautebin L. (2006). Inhibition of nitric oxide biosynthesis by anthocyanin fraction of blackberry extract. *Nitric Oxide*.

[21] Palikova I., Valentova K., Oborna I., Ulrichova J. (2009). Protectivity of blue honeysuckle extract against oxidative human endothelial cells and rat hepatocyte damage. *Journal of Agricultural and Food Chemistry*.

[22] Svarcova I., Heinrich J., Valentova K. (2007). Berry fruits as a source of biologically active compounds: the case of *Lonicera caerulea*. *Biomedical Papers*.

[23] Caprioli G., Iannarelli R., Innocenti M. (2016). Blue honeysuckle fruit (*Lonicera caerulea* L.) from eastern Russia: phenolic composition, nutritional value and biological activities of its polar extracts. *Food & Function*.

[24] Jurikova T., Rop O., Mlcek J. (2011). Phenolic profile of edible honeysuckle berries (genus *lonicera*) and their biological effects. *Molecules*.

[25] Jurikova T., Sochor J., Rop O. (2012). Evaluation of polyphenolic profile and nutritional value of non-traditional fruit species in the Czech Republic--a comparative study. *Molecules*.

[26] Rupasinghe H. P., Boehm M. M., Sekhon-Loodu S., Parmar I., Bors B., Jamieson A. R. (2015). Anti-inflammatory activity of haskap cultivars is polyphenols-dependent. *Biomolecules*.

[27] Chaovanalikit A., Thompson M. M., Wrolstad R. E. (2004). Characterization and quantification of anthocyanins and polyphenolics in blue honeysuckle (*Lonicera caerulea* L.). *Journal of Agricultural and Food Chemistry*.

[28] Wojdylo A., Jáuregui P. N. N., Carbonell-Barrachina A. A., Oszmianski J., Golis T. (2013). Variability of phytochemical properties and content of bioactive compounds in *Lonicera caerulea* L. var. kamtschatica berries. *Journal of Agricultural and Food Chemistry*.

[29] Myjavcova R., Marhol P., Kren V. (2010). Analysis of anthocyanin pigments in *Lonicera* (Caerulea) extracts using chromatographic fractionation followed by microcolumn liquid chromatography-mass spectrometry. *Journal of Chromatography A*.

[30] Chen L., Xin X., Lan R., Yuan Q., Wang X., Li Y. (2014). Isolation of cyanidin 3-glucoside from blue honeysuckle fruits by high-speed counter-current chromatography. *Food Chemistry*.

[31] Gazdik Z., Krska B., Adam V. (2008). Electrochemical determination of the antioxidant potential of some less common fruit species. *Sensors*.

[32] Zhang H., Liu J., Li G. (2018). Fresh red raspberry phytochemicals suppress the growth of hepatocellular carcinoma cells by PTEN/AKT pathway. *The International Journal of Biochemistry & Cell Biology*.

[33] Dong H. W., Zhang S., Sun W. G. (2013). *β*-ionone arrests cell cycle of gastric carcinoma cancer cells by a MAPK pathway. *Archives of Toxicology*.

[34] Liu Q., Dong H. W., Sun W. G. (2013). Apoptosis initiation of beta-ionone in SGC-7901 gastric carcinoma cancer cells via a PI3K-AKT pathway. *Archives of Toxicology*.

[35] Liu J. R., Liu Q., Khoury J. (2016). Hypoxic preconditioning decreases nuclear factor *κ*B activity via disrupted in schizophrenia-1. *The International Journal of Biochemistry & Cell Biology*.

[36] Liu J. R., Yuki K., Baek C., Han X. H., Soriano S. G. (2016). Dexmedetomidine-induced neuroapoptosis is dependent on its cumulative dose. *Anesthesia & Analgesia*.

[37] Liu J. R., Baek C., Han X. H., Shoureshi P., Soriano S. G. (2013). Role of glycogen synthase kinase-3*β* in ketamine-induced developmental neuroapoptosis in rats. *British Journal of Anaesthesia*.

[38] Shan L. H., Sun W. G., Han W. (2012). Roles of fibroblasts from the interface zone in invasion, migration, proliferation and apoptosis of gastric adenocarcinoma. *Journal of Clinical Pathology*.

[39] Duthie S. J., Gardner P. T., Morrice P. C. (2005). DNA stability and lipid peroxidation in vitamin E-deficient rats in vivo and colon cells in vitro. *European Journal of Nutrition*.

[40] Serra D., Paixao J., Nunes C., Dinis T. C. P., Almeida L. M. (2013). Cyanidin-3-glucoside suppresses cytokine-induced inflammatory response in human intestinal cells: comparison with 5-aminosalicylic acid. *PLoS One*.

[41] Forester S. C., Choy Y. Y., Waterhouse A. L., Oteiza P. I. (2014). The anthocyanin metabolites gallic acid, 3-O-methylgallic acid, and 2, 4, 6-trihydroxybenzaldehyde decrease human colon cancer cell viability by regulating pro-oncogenic signals. *Molecular Carcinogenesis*.

[42] Babich H., Sedletcaia A., Kenigsberg B. (2002). In vitro cytotoxicity of protocatechuic acid to cultured human cells from oral tissue: involvement in oxidative stress. *Pharmacology & Toxicology*.

[43] Amararathna M., Hoskin D. W., Rupasinghe H. V. (2020). Anthocyanin-rich haskap (*Lonicera caerulea* L.) berry extracts reduce nitrosamine-induced DNA damage in human normal lung epithelial cells. *Food and Chemical Toxicology*.

[44] Yin M. C., Lin C. C., Wu H. C., Tsao S. M., Hsu C. K. (2009). Apoptotic effects of protocatechuic acid in human breast, lung, liver, cervix, and prostate cancer cells: potential mechanisms of action. *Journal of Agricultural and Food Chemistry*.

[45] Hui C., Bin Y., Xiaoping Y. (2010). Anticancer activities of an anthocyanin-rich extract from black rice against breast cancer cells in vitro and in vivo. *Nutrition and Cancer*.

[46] Liu W., Xu J., Wu S. (2013). Selective anti-proliferation of HER2-positive breast cancer cells by anthocyanins identified by high-throughput screening. *PLoS One*.

[47] Nakamura Y., Torikai K., Ohto Y., Murakami A., Tanaka T., Ohigashi H. (2000). A simple phenolic antioxidant protocatechuic acid enhances tumor promotion and oxidative stress in female ICR mouse skin: dose-and timing-dependent enhancement and involvement of bioactivation by tyrosinase. *Carcinogenesis*.

[48] Chen X., Liao Y., Long D., Yu T., Shen F., Lin X. (2017). The Cdc2/Cdk1 inhibitor, purvalanol A, enhances the cytotoxic effects of taxol through Op18/stathmin in non-small cell lung cancer cells in vitro. *International Journal of Molecular Medicine*.

[49] Liu P., Kao T. P., Huang H. (2008). CDK1 promotes cell proliferation and survival via phosphorylation and inhibition of FOXO1 transcription factor. *Oncogene*.

[50] Dai L., Liu Y., Liu J. (2013). A novel cyclinE/cyclinA-CDK inhibitor targets p27(Kip1) degradation, cell cycle progression and cell survival: implications in cancer therapy. *Cancer Letters*.

